# 

**Published:** 1891-07-04

**Authors:** 


### NOTICE TO CORRESPONDENTS.

All MS., Letters, Books for Review, and other matters intended for the Editor should be addressed The Editor, The Lodge, Porchester Square, London, W.

The Editor cannot undertake to return rejected M.S., even when accompanied by stamped directed envelope.

**Notice to Advertisers and Subscribers.**

All Advertisements, Orders for Copies of The Hospital, and Business Communications should be addressed to The Publisher, *not to the Editor*, The Hospital, 140, Strand, W.C.

The Cost of Subscription, post free, is as follows:—Per week, 1½d.; per quarter, 1s. 8d.; per half-year, 3s. 3d.; per annum, 6s. 6d. Foreign:—Per annum, 8s. 8d.; payable, in all cases, in advance.

**NOTICE.—The Index for Vol. IX., (ending March, 1891) is on sale at the Office of this Journal, price 2½d., post free.**

### GENERAL CHARITIES.

[This Column is devoted to an account of work of charitable institutions other than Medical Charities. The Editor will be glad to receive reports or news of work at 140, Strand. *Philanthropy* should be plainly written on the envelope.]

The total subscriptions at the **NEWSPAPER PRESS FUND DINNER** amounted to £1,200.

Mr. William Denham has been appointed the secretary of the **HOMES FOR WORKING BOYS**, at the office in Buckingham Street.

**THE ROYAL ALFRED AGED MERCHANT SEAMEN'S INSTITUTION** reaped a splendid subscription at the annual festival; Sir James Anderson, the chairman, gave £500, and the sum total amounted to £4,107.

The **ANNUAL FESTIVALS** of following institutions are fixed: The Homes for Little Boys at Farningham and Swanley, on Saturday, July 18th; the London Orphan Asylum, Watford, on Saturday, July 11th; the Royal Asylum of St. Anne's Society's Schools, at Salters' Hall, on Tuesday, July 7th; the British Orphan Asylum, Slough, on Saturday, July 11th.

Two largely-attended meetings were held last Saturday of supporters of **THE SOCIETY FOR THE PREVENTION OF CRUELTY TO ANIMALS.** The sixty-seventh annual meeting was presided over by Lord Aberdare, and the report presented to the meeting has both its satisfactory and unsatisfactory features. It appears that horses, donkeys, ducks, geese, and turkeys were treated more humanely during 1890 than 1889, but that cattle, sheep, pigs, fowls, and wild birds were not so fortunate. As many as 5,890 people had been punished through the agency of the society during the year, an eloquent but painful testimony to the necessity of its existence. In the afternoon the Duchess of Portland distributed prizes to those scholars of metropolitan schools who had written the best essays on the subject of kindness to animals.

The seventy-third annual meeting of **THE MENDICITY SOCIETY** was this year presided over by the Duke of Wellington. We are very sorry to see there is a little falling off of donations to this Society, which has a record of work enough to satisfy the most exacting subscribers. During the year 912 adult vagrants have been apprehended, 6,966 food tickets have been presented, and 1,287 begging letters have been reported on. It is important to remind the public that the chief work of the Society consists in employing a body of men capable of making themselves acquainted with the life, habits, and tricks of professional mendicants, and, after bringing the latter before the magistrates, in assisting them to earn an honest living on their discharge from prison. The Queen, who places all her begging letters in the hands of this Society, has increased her subscription to £100. "Agencies like the Mendicity Society," said Lord Selborne, "are more valuable now than they have ever been, at a time when emotion is obtaining such a control over society in every way." It is just this emotion that stands in the way of necessitous poverty so much—the sturdy beggar flourishes, and genuine want starves unnoticed.

Sir Michael Hicks-Beach, M.P., President of the Board of Trade, took the chair at the fifty-second annual meeting of **THE NATIONAL MARITIME RELIEF ORGANIZATION**, held at the Hotel Metropole, Charing Cross, on June 17. The annual report, read by the Secretary, Mr. W. R. Buck, stated at the outset that as a consequence of the more stormy weather of 1890, as compared with 1889, the wreck returns for the year showed an increase in the annual figures. In view of the society's operations in the charitable care of the rescued, as well as of the distressed families of those lost, it was noted that by the latest-issued Board of Trade Returns, 8,519 gravely imperilled lives were saved, in one year, by various means, not infrequently at great risk and in destitute condition, from shipwrecked vessels of all kinds, at home and abroad. Keeping pace with the greater annual number of sea disasters, the society's relief statistics exhibited an increase of the shipwrecked and bereaved urgently assisted in their extremity, in 1890—namely, under this one head alone, a total of 5,311 sufferers, as against 3,706 in 1889. While assistance of the shipwrecked, with prompt seeking out and relieving of the widow and the orphan, had been the society's first care, its benevolent functions were extended within the past year to very numerous other cases of claim and need amongst the seafaring classes—the aggregate numbers assisted, under all heads, in 1890, representing the large total of 10,345 persons, as against 9,285 in 1889, and bringing up the numbers relieved by the society, since it was first instituted in 1839, to a grand total of 426,447 persons. The financial statement showed the receipts within the past year to have been £20,995, and the expenditure £21,553, the former including over £6,000 providently contributed by mariners and fishermen themselves, under the society's thrift section. The noteworthy fact was further recorded that, commencing from a single half-crown, the society had, during the fifty-two years of its charitable existence, received from all sources the total amount of no less than £919,875.

### SCRAPS AND GLEANINGS.

BOSTON HOSPITAL has received a legacy of £300.

HOSPITAL Saturday at Lincoln brought in £182.

LUTON Hospital Saturday Fund has reached £92.

THE Hospital Sunday Fund has reached £39,000.

THE Sunderland Saturday Collection has reached £257, being £25 over that of last year.

SALOP Infirmary has decided that its surgeons must retire when they reach the age of 62.

FOURTEEN cases of sunstroke, four of them fatal, were reported in Berlin on Saturday.

CHOLERA has broken out in the village of Kili, in the Starim district of the Vilajet of Aleppo.

THE Prince and Princess of Wales visited the Princess Alice Hospital while they were at Eastbourne.

THE Public Health Bill has made such progress that there is no doubt now that the measure will pass into law.

EARL SPENCER presided on Tuesday night at the Middlesex Hospital, and gave the prizes to the successful students.

A CHARMING amateur dramatic entertainment in aid of St. Elizabeth's Hospital was given at the house of Lady Alexander Gordon-Lennox last week.

IT is asserted on the authority of a well-known skin specialist that instances of skin disease contracted in barbers' shops are multiplying with alarming frequency.

THE Home Visiting question at Guildford Hospital has been settled by a compromise, the Committee undertaking to carry on the home visiting for another five years, and then to be free to drop it.

A REUTER'S telegram from Bombay says that the Nizam's Government have subscribed £100 towards the expenses of the International Congress of Hygiene and Demography, to be held in London.

PRINCESS CHRISTIAN presented, at the Albert Institute, Windsor, last week, the medallions and certificates gained by the members of the Windsor and Cumberland Lodge branches of the St. John Ambulance Association.

MR. W. H. SMYTH presided at the annual meeting of subscribers to Louth Hospital. The treasurer's report showed an adverse balance of £96 19s. 7d., there having been heavy expenses in connection with alteration to the drainage system.

AN apothecary named Bombel, of Neuenhaus, claims to have discovered a process whereby the lymph known as Dr. Koch's may be purged of its dangerous qualities. His experiments with the lymph so purged are said to have met with great success.

AT a meeting of the Commissioners of Sewers, held in the Guildhall, last week, it was resolved, on the motion of Mr. Malthouse, to instruct the Sanitary Committee "to consider and report upon the desirability and cost of erecting a crematorium at the cemetery at Ilford.

AN outbreak of rabies has occurred in Carlow among the deer at Oak Park, and the animals destroyed exceed 100 head. Before the rabid dog attacked the deer it bit three children, who have been sent to the Pasteur Institute at Paris for treatment at the expense of the Carlow Guardians.

SOUTHAMPTON Hospital Sunday Fund has been apportioned as follows: Royal South Hants Infirmary, £788 11s.; Southampton Dispensary, £105 8s.; Nurses' Training Institute, £37 6s.; St. Mary's Cottage Hospital, £64 13s.; Shirley Cottage Hospital, £23 4s.; Southampton Maternity Society, £7 11s.; total, £1,026 13s.

THE visit of the Prince and Princess of Wales to St. Mary's Hospital, fixed for July, will not now take place till next summer, as they have acceded to the request of the hospital authorities that they would postpone their visit till the arrangements for laying the foundation-stone of the new buildings are in a more forward state.

THE High Sheriff of Lincolnshire, Mr. Joseph Ruston, on Saturday afternoon laid the foundation-stone of a new building at the County Hospital at Lincoln. A want has long been felt at the hospital for a children's ward and an eye ward, and Mr. Ruston generously came forward and offered to make the necessary additions to the hospital buildings at his own expense.

IT is stated that the Austrian Minister of the Interior has recently issued an ordinance that the burgomasters of all communes must exercise strict supervision over the medical men practising within their jurisdiction in the matter of legibility of prescriptions. They are charged to see that every prescription is clearly and legibly written in all its parts, so that there may be no doubt as to the remedy, the dose, or the signature.

THE late Miss Mary Bradford bequeathed the following legacies, viz.: To Owens College, Manchester, £5,000; to the Northern Counties Hospital for Incurables, £5,000; to the Manchester City Mission, £5,000; to the Church Missionary Society, £5,000; to the Manchester Royal Infirmary, £2,000; to the St. Mary's Hospital and Dispensary, £2,000; to the Governesses' United Kingdom Beneficent Society, London, £2,000; to the Clinical Hospital and Dispensary for Children, Cheetham, £1,000; and to the Boys' Refuge, Strangeways, £1,000.

MR. DAVID PRICE has left £2,000 each to the Metropolitan Hospital, the London Fever Hospital, the Royal Free Hospital, the London Hospital, the North London Consumption Hospital, the National Hospital for the Paralysed and Epileptic, the City of London Hospital for Diseases of the Chest, the Small-Pox and Vaccination Hospital, the Middlesex Hospital, the Hospital for Incurables, the Free Cancer Hospital, University College Hospital, the Royal London Ophthalmic Hospital, the West-End Hospital for Diseases of the Nervous System and Paralysis and Epilepsy.

### The Student of Medicine.

[All communications for this Supplement should be addressed to The Editor of The Hospital, at 140, Strand, with the words, "Students' Column," written in the left hand top corner of the envelope.]

### LEADING ATHLETES.

We give this week the sketch of the athletic career of Mr. G. S. S. Marshall, who is best known in the "running" world, but also of considerable renown at cricket and at Rugby football. He was educated at Newent Grammar School, and at St. Andrew's College, Chardstock, and afterwards entered at the Middlesex Hospital. He first started running in June, 1888, but only ran a few times in that year. In the following year he was very successful, winning, among other events, the 100 yards and the 220 yards at the United Hospital Sports. Also in the match against Edinburgh the same year he ran "dead heat" in the 100 yards with the Scottish amateur champion. In 1890 he ran but seldom, and, owing chiefly to indisposition, was not very successful, but in the match against the L.A.C. he ran "dead heat" with Pelling and Green. He also obtained fourth place in the 100 yards amateur championship, being the only Southerner in the final. Since he has been on the path Mr. Marshall has won thirty-two prizes, and also some prizes at lawn tennis. As a cricketer he is a good bat and a good bowler. In 1888 he played for the Gloucestershire Colts against the county, and scored 72 against the county bowling, but for some unknown reason he was never tried for the county. At football he is one of the best three-quarter backs in London, in which position he has played in a great number of important matches for his hospital, for the United Hospital Club, and also for Gloucester and the Western Counties, having played for the last against London and Middlesex. At the present time Mr. Marshall is captain both of the Middlesex Hospital cricket and also of the football clubs, and hon. secretary of the United Hospitals Rugby Football Club, and hon. treasurer of the United Hospitals Athletic Club.

### APPOINTMENTS.

At Guy's Hospital the following appointments have been made:—J. H. Bryant, M.B., B.S.Lond., house physician; C. Mc. G. Kitching, M.B., B.S.Lond., house physician; T. G. Stevens, M.B., B.S.Lond., house surgeon; F. Wellford, M.B.Camb., house surgeon. At the Dewsbury and District Infirmary, J. W. Applegate, L.R.C.P., M.R.C.S., has been appointed house surgeon. At the West of England Institution for Deaf and Dumb Children, Russell Coombe, M.A.Camb., F.R.C.S., has been appointed surgeon. At the Croydon General Hospital, W. H. Drew, F.R.C.S., has been appointed surgeon. At the General Hospital for Sick Children, Pendlebury, Manchester, A. W. W. Lea, M.B., B.S.Lond., has been appointed resident medical officer; and F. H. Westmacott, M.R.C.S., L.R.C.P., senior resident medical officer. At the Royal Infirmary Aberdeen, Alexander Macgregor, M.B., C.M.Aberd., has been appointed medical electrician. At St. Mary's Hospital, Morton Smale, M.R.C.S., L.D.S., L.S.A., has been appointed dental surgeon.

### VACANCIES.

The following vacancies are announced this week, the amount of salary in each case being given after the name of the institution:

### Resident Medical Officers.

Durham County Hospital, House Surgeon—Salary £100, and board, &c.
General Infirmary at Gloucester and Gloucestershire Eye Institution, House Surgeon—Salary £100, and board, &c.
Hospital for Consumption and Diseases of the Chest, House Physicians—Board, &c.
National Sanatorium for Consumption and Diseases of the Chest, Bournemouth, House Surgeon and Secretary—Salary £120, and board, &c.
Tunbridge Wells General Hospital, House Surgeon—Salary £100, and board, &c.
Western General Dispensary, Marylebone Road, second House Surgeon—Salary £50, and board, &c.

### Non-Resident Medical Officers.

Glasgow Maternity Hospital, Out-door Physician for the South Eastern District.
Queen's College, Birmingham, Medical Tutor and Demonstrator of Anatomy.
St. Thomas's Hospital Medical School, Demonstrator of Physiology.

### THE STUDENT OF MEDICINE—(*continued*).

### PASS LISTS.

**Cambridge University.**

FIRST EXAMINATION FOR MEDICAL AND SURGICAL DEGREES. EASTER TERM, 1891.

PART II.—ELEMENTARY BIOLOGY.

EXAMINED AND APPROVED: Adams, Pet.; Auden, Christ's; A. M. Barraclough, H. Cav.; Betteridge, Cai.; Blatchford, Sid.; Bourne, H. Cav.; G. F. Briggs, Joh.; Bytheli, Joh.; Carter, Jesus; Charlton, Cai.; Collingwood, Cai.; Crawley, Christ's; de Havilland, B.A., Pet.: Dove, Christ's; Edmondson, Clare; Fletcher, Cai.; Gaine, Emman.; Gardiner, Trin. H.; L. S. Gaskell, Christ's; Gillett, Down; Gilmore, Christ's; Graham, Trin.; Green, Down; Grosvenor, Trin.; Halahan, Corpus; L. K. Harrison, Cai.; A. E. Harrison, Magd.; Hawkins, Trin; Holmes, Joh.; Howitt, Cai.; E. Hyde, Clare; W. M. James, Christ's; Jephcott, Cai.; Judd, non-collegiate; E. B. H. Kershaw, Joh.; King, H. Selw.; Langdon, Cai.; Lees, Cai.; E. T. Longhurst, H. Aye.; Lord, Joh.; M'Dougoll, Joh.; MacGregor, Trin.; Mathias, Christ's; Moritz, Cai.; Naish, Trin.; G. B. Nicholson, Clare; Parker, Cai.; Parsons, Corpus; Paterson, Emman.; Pendlebury, Pemb., Prince, Cai.; Rae, Joh.; Rawling, Cai.; Reid, Joh.; Rigby, Cai.; Roberts, B.A.; Down; R. S. Rowland, Corpus; Salt, Emman.; Sapwell, Cai.; Scaping, Clare; Selby, Down; Senior, H. Cav.; Shoyer, Trin. H.; C. C. Simson, Trin.; Sing, Christ's; W. C. P. Smith, H. Cav.; Stawell, Trin. H.; Sturrock, Cai.; Thomas, Cai.; Tuckett, Trin.; F. F. Ward, H. Selw.; Watson, Trin.; F. K. Weaver, Trin.; A. U. Whittingham, Trin. H.; Wilkinson, Emman; Woodhouse, King's; Woolley, Cai.; Woolley, Christ's; Young, Cai.

SECOND EXAMINATION FOR MEDICAL AND SURGICAL DEGREES. EASTER TERM, 1891.

PART II.—HUMAN ANATOMY AND PHYSIOLOGY.

EXAMINED AND APPROVED.—Appleyard, B.A., Emman.; Atkinson, B.A., Clare; Bowes, Cai.; Burrell, B.A., Cai.; Cotter, B.A., Trin.; H. J. Davis, B.A., Trin; Delbruck, B.A., King's; Ferguson, B.A., Cai.; L. N. Harding, B.A., H. Selw.; Hayne, Cai.; A. B. Heaton, B.A., Trin.; Henry, B.A., Joh.; Hood, H. Cav.; C. L. Hopkins, Cai.: Menzies, Jesus; Mitchell, B.A., Queen's; Moysey, B.A., Cai.; Nachbar, B.A., Clare, Norris, B.A., Christ's; Ormerod, B.A., Trin.; Peck, B.A., Trin.; Peters, B.A., Caius; Petyt, B.A., Christ's; Sandall, Joh.; Seccombe, B.A., Joh.; H. Smith, B.A., Trin.; Todd, Clare; Trethewy, Cai.; White, B.A., Sid; Wrangham, B.A., Emman.

**Oxford.**

The following degrees have been conferred:—

M.D.—Arthur M. Jackson, Queen's; Frederick A. Dixey (Fellow), Wadham.

M.B.—Edward T. Withington, Balliol; Alexander B. Roxburgh, Exeter; George Jones, Oriel; Alfred M. Gossage, Magdalen; Charles P. Lovell, St. John's; Lewis E. Parkhurst, Jesus; Edward J. Moore, Christ Church; Frederick H. Burton-Browne, Magdalen.

**Royal College of Surgeons of England.**

The following gentleman having passed the necessary examination, and having conformed to the by-laws and regulations, was, at an extraordinary meeting of the Council, on Thursday, the 25th inst., admitted a Member of the College: Frederick James Brown, L.S.A., Bradford Street, Bolton.

### DISTRIBUTION OF PRIZES.

THE distribution of prizes and scholarships to the successful students of the Middlesex Hospital took place on Tuesday last, the 30th ult.; Earl Spencer K.G., was in the chair; and at Charing Cross Hospital, on Wednesday, July 1st., the Right Hon. the Lord Cunningham presided.

The distribution at Guy's Hospital has been fixed for Wednesday, July 8th, at 3.30 p.m., when J. A. Shaw Stewart, Esq., will preside.

### ATHLETICS.

**United Hospitals' Athletic Sports.**

On Tuesday, June 23rd, the annual meeting of the above club took place in weather that was not quite so agreeable as it might have been, but, nevertheless, everything went off in great style. The attendance on the whole was good, and much enthusiasm was displayed by the visitors. On this occasion several of the previous records of these meetings were beaten, the lion of the day being B. C. Green, of Bart's., whose athletic career we published a few weeks ago, and he has already added so much to that account there will soon be material enough for another sketch of his winnings. In the sprint he won easily, and if he had been pressed would probably have done still better; as it happened he improved on his last year's time by 1-5th of a second. In the hurdle race he was again easily first, and was within a very small fraction of Granby's record. He also won the 220 yards and the running broad jump. The broad jump was a wonderful performance, the three first men jumping over 21 feet, and four beating the jump of the holder for the previous year. H. A. Monro, of Guy's, also won in grand style both the mile and the three mile races. The winning events by the several hospitals was as follows: St. Bartholomew's six firsts and two seconds, Green obtaining three firsts and one second, M'Intosh, Richards, and Hay a first each, and Wilde a second; Guy's, two firsts and one second, Monro getting two firsts and Bell the second; Middlesex gained likewise three prizes, Marshall and Square taking the two firsts and Marshall also a second; St. Mary's, two prizes, Langton taking a first and a second; London took a first with Rolfe; Breton, for St. George's, obtained two seconds; and M'Cullock, of Charing Cross, and Wace, of King's, gained a second each for their respective hospitals. The following were the officials: Judges, A. F. Voelcker, M.D., G. R. Turner, F.R.C.S., and H. M. Fletcher, M.B.; referee, A. Bowlby, F.R.C.S.; timekeeper, F. Furnivall; starter, C. L.

### THE STUDENT OF MEDICINE—(*continued*).

Lockton; clerks of the course, W. Kent Hughes and A. E. Madge. Particulars of the events:—

100 Yards Run (challenge cup; holder, B. C. Green, St. Bartholomew's. Time, 10 3-5 sec. First in each heat, and the second in the fastest heat, to run in the final.)—Heat 1: B. C. Green, St. Bartholomew's, first; J. Stokes, second; G. E. Webster, St. Bartholomew's, 0; E. M. Hetherington, King's, 0; E. W. Beasley, St. Mary's, 0. An easy win. Time, 10 4-5 sec. Heat 2: G. S. S. Marshall, Middlesex, first; J. W. Summerhayes, St. Mary's, second; H. T. Roberts Charing Cross, 0; L. A. Hill, St. Thomas's, 0. Won by a yard. Time, 10 4-5 sec. Heat 3: H. T. Bell, Guy's, first; A. P. Square, Middlesex, second; R. H. Steinhaeuseur, Guy's, 0; G. Hancock, Westminster, 0; M. Breton, St. George's, 0. Won easily. Time, 10 4-5 sec. Final heat: Green, first; Marshall, second; Summerhayes, third. Won by a yard; with four feet separating second and third. Time, 10 2-5. The best record ever made at these games was 10 1-5 sec., made by G. B. Connelley in 1868.

Half-Mile Run (Challenge Cup—holder, A. R. Badger, St. Bartholomew's. Time, 2 min. 6 3-5 sec.).—A. Hay, St. Bartholomew's, first; A. R. McCullagh, Charing Cross, second; H. Bailey-King, Middlesex, third; F. M. Gilmore, St. Thomas's, 0; J. W. A. Cooper, King's, 0; H. W. Dudgeon, Guy's, 0; P. G. Mould, St. Mary's, 0; H. G. Waters, St. Thomas's, 0; H. J. Spon, Guy's, 0; W. J. Richards, St. Bartholomew's, 0; A. L. Williams, Middlesex, 0; J. C. Gardner, St. George's, 0. This was a magnificent race, the first two men alternating in the lead until the last hundred yards, when Hay spurted to the front, and won by a couple of yards, with only two feet parting second and third. Time, 2 min. 4 1-5 sec. The best time made at Hospital Sports was Kent-Hughes, St. Bartholomew's, 2 min. 2½ sec.

Putting the 10lb. Shot (holder, H. G. West, St. Bartholomew's, distance, 36ft. 4½in.).—C. Rolf, London Hospital. 34ft. 10in., first; M. Breton, St. George's, 33ft. 7½in., second; J. S. Mackintosh, St. Bartholomew's, third; S. Langton, St. Mary's, 0; J. L. Molloy, Charing Cross, 0; S. Lee, Middlesex, 0; E. Moore, Guy's, 0; W. K. Hopkins, St. Bartholomew's, 0; E. Bromet, St. Thomas's, 0; F. W. Robinson, Guy's, 0; and H. Charles, Middlesex, 0. The record for Hospital Sports is 38ft. 3in., held by W. G. West, St. Bartholomew's. In an exhibition put, M. Breton, the second man, sent the iron sphere 35ft., but, unluckily for him, it did not count in the regular competition.

120 Yards Hurdle Race (holder, B. C. Green, St. Bartholomew's; time, 17 sec.); first in each heat, and the second in the fastest heat to run in the final).—Heat 1; G. W. Thompson, St. Thomas's, first; R. Herschell, Middlesex, second; E. H. Steinhaeuseur, Guy's, 0; H. J. Spon, Guy's, 0. Won easily. Time, 18 4-5 sec. Heat 2: B. C. Green, St. Bartholomew's, first; S. Langton, St. Mary's, second; G. S. S. Marshall, Middlesex, 0; S. Kent, St. Bartholomew's, 0. An easy win. Time, 17 sec. Final Heat: Green, first; Langton, second; Thompson, third. The winner went loose from the field at the first rise, and running away at every stride, won easily by three yards. The race for second place was a keen one, and only a couple of feet parted Langton from Thompson at the finish. Time, 16 4-5 sec. This is only a few inches worse than J. G. Graveley's record of 16¾ sec. made four years ago.

Running Broad Jump (holder, R. G. Hogarth, St. Bartholomew's, 19 ft. 8 in.).—B. C. Green, St. Bartholomew's, 21 ft. 10 in., first; A. P. Square, Middlesex, 21 ft. 3¾ in., second; G. Thompson, St. Thomas's; 21 ft., 1 in., third; H. T. Bell, Guy's, 19 ft. 10 in., fourth; G. M. Hetherington, King's, 19 ft. 3 in., fifth; J. G. Barron, St. Bartholomew's, 0; R. Herschell, Middlesex, 0; G. F. Baxter, Westminster, 0; E. J. Goffe, University, 0. Green's jump supplants R. G. Hogarth's record of 21 ft. 2 in.

220 Yards Run (holder, B. C. Green; time, 23 1-5 sec.)—.—The schedule called for two heats, but there were so many absentees that it was decided to run the affair off in one dash. B. C. Green, St. Bartholomew's, first; G. S. S. Marshall, Middlesex, second; L. A. L. Hill, St. Thomas's, †; J. W. Summerhays, St. Mary's, †. Again Green had an easy win, but Marshall had a close shave for second position; Hill and Summerhays made a dead heat for third place. Time, 23 sec. G. S. S. Marshall, Middlesex, holds the record for this distance at 22 4 5 sec.

Throwing the 16 lb. Hammer (holder, J. E. Fraser, St. Bartholomew's Hospital; distance, 93 ft. 10 in.; this also proves the best on record for these sports). J. S. Mackintosh, St. Bartholomew's, 89 ft. 11 in., first, M. Breton, St. George's, 85 ft. 4½ in., second; J. L. Molloy, Charing Cross, 0.

Mile Race (Challenge Cup; holder, C. Wace, King's College. Time, 4 min. 38 1-5 sec.)—H. A. Munro, Guy's, first; C. Wace, King's, second; —. James, St. Bartholomew's, third; H. B. Ellerton, St. Mary's, fourth; H. Bailey King, Middlesex, 0; A. Hay, St. Bartholomew's, 0. Munro jumped off in the lead, and set the pace for a furlong, when he dropped back, allowing Wace to become the pioneer. At the end of the first lap the order of going was Wace, Munro, James, with a yard between each of them. During the second lap Munro again went to the front, leading Wace a couple of yards at the end of the half-mile. In the third lap Munro spurted, and the race was never afterwards in doubt, as the L.A.C. man, travelling in good style, opened out a big gap, and eventually won easily by thirty yards in 4 min. 31 1-5 sec., which supplants H. P. Ward's (King's College) record of 4 min. 36 4 5 sec. Wace beat James by 175 yards for the place, and Ellerton was tailed off.

Running High Jump (holder, P. Pierre, Westminster, 5 ft. 2½ in.).—S. Langton, St. Mary's, 5 ft. 5½ in., first; H. T. Bell, Guy's, 5 ft. 4½ in., second; G. Elliott, University, 5 ft. 3½ in., third; J. H. R. Bond, St. Mary's, 5 ft. 1¼ in., fourth; M. E. Tressider, Guy's, 4 ft. 11½ in., fifth. The jumping was started at 4 ft. 6 in., and the bar was raised two inches at a time until Tressidder went out; then the bar was raised one inch at the time. Bell, the smallest of the jumpers, got over the bar in the neatest fashion, but Langton was much stronger than his antagonists.

Quarter-mile Run (Challenge Cup; holder B. C. Green, St. Bartholomew's; time, 53 2-5 sec.).—The programme called for two heats, but

### THE STUDENT OF MEDICINE—(*continued*).

the event was run off in one dash, starting at the top of the straight course. W. G. Richards, St. Bartholomew's, first; B. C. Green, St. Bartholomew's, second; P. G. Mould, St. Mary's, third; J. J. Hamilton, King's, 0; J. W. Summerhayes St. Mary's, 0; W. H. Lucas, Middlesex, 0. Won by two yards; Mould came very strongly at the finish, Green beating him by three yards only. Time, 53 1-5 sec. T. A. Guinness King's, holds the record at 51 4 5 sec.

Three Miles Run (Challenge Cup); holder. A. Quennell, St Bartholomew's; time, 16 min. 19 2-5 sec.).—H. A. Munro, Guy's, first; A. N. Wilde, St. Bartholomew's, second; T. W. A. Cooper, King's, third; C. H. Nicholson, Middlesex, 0; H. Knight, St. Thomas's, 0; M. E. Tressider, Guy's, 0. Won easily by over fifty yards. Time, 15 min. 47 2-5 sec. Wilde got home in 16 min. 1 4-5 sec., with Cooper third, 150 yards behind. The winner's time beat A. Quennell's record of 15 min. 52 sec.

220 Yards Open Handicap.—Heat 1: R. M. Dean, 20 yards start, first; W. E. Newbeggin, 17, second; C. M. Woodward, 17, third; F. S. Wayland, 13, 0. Won by four feet, with half that distance separating second and third. Time, 22 3 5 sec. Heat 2: D. Basan, 2, first; G. K. House, scratch, second; J. P. Shuter, 3, third; B. Robinson, 12, 0. Won by two feet only; there was only a foot between second and third. Time, 22 2-5 sec. Final heat: Basan, first; Dean, second; House, third. Won by two feet, with the same distance separating second from third. Time, 22 2-5 sec.

### Cricket.

*The Inter-Hospital Cup Tie*.—At Richmond Cricket Ground, on Monday, the 22nd, the semi final of the inter-hospital cup tie was played by the representatives of the Charing Cross Hospital and those of King's College. Some heavy scoring took place on the part of the Charing Cross men—Wright making 85, Escombe 54, Agnew 45, Russel 32, and Thomas 16, the result of the whole innings amounting to 276 runs. On the side of King's College the highest scorers were—Robardt 23, and Adams 16. None of the other players reached double figures, and the innings terminated with a score of 92 runs. This result promises well for Charing Cross Hospital players in the final cup tie, which has to be played against Guy's Hospital.

*Charing Cross Hospital v. Redhill.*—Played at Redhill, June 20th, and resulted in a win for the Hospital by 12 runs. Score: Redhill—J. Coates 44, W. Edwards 3, H. Trower 13, J. Maude 14, G. Coomber 33, T. Cooke 4, W. Hughes 18, E. A. Philpott 9, C. R. Gear 2, E. Puplett 0, W. Shore, not out, 0; byes, &c., 26; total, 166. Charing Cross Hospital—C. S. Agnew 10, C. E. Young 4, C. Forsyth 12, W. Paton 18, C. Lanphier 68, S. Wallace 9, H. H. Thomas 13, M. T. Archdale 0, W. A. Stephenson 22, E. C. Montgomery, not out, 5; S. R. Merry 6; byes, &c., 11; total, 178.

On Saturday, June 27th, *Middlesex Hospital* were beaten by *High Wycombe*, at Wycombe, by 101 runs on the first innings. Score: Middlesex, 91 and 146 (for six wickets); Wycombe, 192.

### United Hospital Rowing Club.

*Inter-Hospital Challenge Cup.*—The annual four race for this cup was rowed on Friday, June 26th, between the crews representing the various metropolitan hospitals, over the usual course from Putney Bridge to Hammersmith Bridge. This is the seventh year that this magnificent cup has been rowed for, the competition being started in 1885, and has now become one of the great events of the year among rowing men. The first race in 1885 was won by London, the two following years the cup was held by Middlesex, then for two years by St. Thomas's, and last year by St. George's. This season the holders, St. George's, on account of the unavoidable absence of their renowned light blue stroke, J. C. Gardner, who is training with the Thames Rowing Club for the Henley Regatta, did not compete. London, the winning crew, although they made a bad start, won easily, and appeared to be much better trained than any of the others, having been coached by W. Gibson, one of the famous aquatic and fisher family of Putney. London, Middlesex, and St. Thomas's sent crews. At the word Middlesex were first away, followed by St. Thomas's, the third crew, London, doing very badly at first, their No. 3 catching a "crab," putting them all out. On settling down, however, London came up hand over hand, and securing the lead at the Dummy headed the others, who were level three quarters of a length off Alexander's. Reaching Bishop's Creek in 1 min. 40 sec., a length separated London and Middlesex, with St. Thomas's half a length further in the rear. Arriving at the top of the New Embankment the difference was half a length clear, with the other two level, and thence the leaders gained rapidly. Passing Craven steps in 3 min. 5 sec., London were a length clear of St. Thomas's, who just headed Middlesex, and the first-name going right away from the Crab Tree, finally won by several clear lengths, St. Thomas beating Middlesex, after a good race, by a couple of lengths. Time, 9 min. 5 sec., there being a fair flood.

Surrey Station: London: H. W. Evans (bow), 10 st.; 2, J. Moses, 12 st. 1 lb.; 3, W. Bird, 12 st. 2 lb.; G. Boyd (stroke), 10 st. 4 lb.; E. C. Smith (cox.), 8 st. 3 lb. .......... 1

Middlesex Station: St. Thomas's: R. E. Nix (bow), 11 st.; 2, J. Carver, 11 st. 7 lb.; 3, H. J. Davis, 12 st. 10 lb.; T. H. Haydon (stroke), 10 st. 10 lb.; W. Allen (cox.), 9 st. 5 lb. .......... 2

Centre Station: Middlesex: L. A. Williams (bow), 10 st. 7 lb.; 2, C. E. Maude, 11 st. 4 lb.; 3, A. F. Palmer, 12 st. 4 lb.; C. H. Nicholson (stroke), 11 st. 7 lb.; W. H. Johnson (cox.), 8 st. 5 lb. .......... 3

WINNERS SINCE THE FOUNDATION.

| | | |
|---|---|---|
| 1885. London. | 1888. St. Thomas's. | 1890. St. George's. |
| 1886. Middlesex. | 1889. St. Thomas's. | 1891. London. |
| 1887. Middlesex. | | |

At the Amateur Athletic Championship meeting at Manchester on June 27th, E. Frazer (Bart's.) obtained a silver medal for throwing the hammer, the throw being 106 ft. 8 in.

